# Microbial Biotransformation of a Polyphenol-Rich Potato Extract Affects Antioxidant Capacity in a Simulated Gastrointestinal Model

**DOI:** 10.3390/antiox7030043

**Published:** 2018-03-20

**Authors:** Joelle Khairallah, Shima Sadeghi Ekbatan, Kebba Sabally, Michèle M. Iskandar, Raza Hussain, Atef Nassar, Lekha Sleno, Laetitia Rodes, Satya Prakash, Danielle J. Donnelly, Stan Kubow

**Affiliations:** 1 School of Human Nutrition, McGill University, 21111 Lakeshore, Ste. Anne de Bellevue, QC H9X 3V9, Canada; joelle.khairallah@mail.mcgill.ca (J.K.); shima.sadeghi@mail.mcgill.ca (S.S.E.); kebba.sabally@mcgill.ca (K.S.); michele.iskandar@mail.mcgill.ca (M.M.I.); raza.hussain@mail.mcgill.ca (R.H.); atef.nassar@mail.mcgill.ca (A.N.); 2 Plant Science Department, McGill University, 21111 Lakeshore, Ste. Anne de Bellevue, QC H9X 3V9, Canada; danielle.donnelly@mcgill.ca; 3 Chemistry Department, University of Quebec at Montreal, 2101 rue Jeanne-Mance, Montreal, QC H2X 2J6, Canada; sleno.lekha@uqam.ca; 4 Department of Biomedical Engineering, Duff Medical Building, McGill University, 3775 Rue University, Montreal, QC H3A 2B4, Canada; laetitia.rodes@mail.mcgill.ca (L.R.); satya.prakash@mcgill.ca (S.P.)

**Keywords:** phenolic metabolites, *Solanum tuberosum*, biotransformation, polyphenols, potato, antioxidant, digestion, gastrointestinal model

## Abstract

A multistage human gastrointestinal model was used to digest a polyphenol-rich potato extract containing chlorogenic acid, caffeic acid, ferulic acid, and rutin as the primary polyphenols, to assess for their microbial biotransformation and to measure changes in antioxidant capacity in up to 24 h of digestion. The biotransformation of polyphenols was assessed by liquid chromatography–mass spectrometry. Antioxidant capacity was measured by the ferric reducing antioxidant power (FRAP) assay. Among the colonic reactors, parent (poly)phenols were detected in the ascending (AC), but not the transverse (TC) or descending (DC) colons. The most abundant microbial phenolic metabolites in all colonic reactors included derivatives of propionic acid, acetic acid, and benzoic acid. As compared to the baseline, an earlier increase in antioxidant capacity (*T* = 8 h) was seen in the stomach and small intestine vessels as compared to the AC (*T* = 16 h) and TC and DC (*T* = 24 h). The increase in antioxidant capacity observed in the DC and TC can be linked to the accumulation of microbial smaller-molecular-weight phenolic catabolites, as the parent polyphenolics had completely degraded in those vessels. The colonic microbial digestion of potato-based polyphenols could lead to improved colonic health, as this generates phenolic metabolites with significant antioxidant potential.

## 1. Introduction

Dietary (poly)phenolic compounds are secondary metabolites widespread in fruits, vegetables, seeds, and plant-derived beverages such as tea and coffee [1]. Among the wide variety of (poly)phenolics found in foods, phenolic acids and flavonoids have received the greatest research attention for their antioxidant, antiobesity, anti-inflammatory, and antidiabetic properties [2,3,4]. Potatoes contain significant amounts of (poly)phenolics including phenolic acids as well as flavonols such as rutin [5,6], in addition to other bioactive phytochemicals including glycoalkaloids [7,8]. Due to their high consumption as a food staple, potatoes (*Solanum tuberosum* L.) are an important dietary source of (poly)phenolics in the French, American, and Greek diets [9,10]. A (poly)phenolic-rich potato extract (PRPE), containing chlorogenic acid, caffeic acid, ferulic acid, and rutin as the major (poly)phenolic constituents, has been demonstrated to protect against glucose intolerance and obesity in male and female mice fed a high-fat diet [11].

Since (poly)phenolics are present in foods as esters, polymers, or glycosides, they are generally poorly absorbed in the upper gastrointestinal (GI) tract [12,13,14]. For example, only one-third of ingested chlorogenic acid and 2–15% of flavonoids were estimated to be absorbed in the stomach and small intestine (SI) [15]. Due to their poor bioavailability, substantial amounts of (poly)phenolics reach the large intestine, where they undergo degradation by gut microflora into a range of smaller-molecular-weight compounds [16]. The gut microbial-mediated biotransformation of (poly)phenolics could contribute to their systemic health benefits, as their smaller-molecular-weight by-products are more bioavailable [17]. This phenomenon is evidenced by the appearance of microbial metabolites in the plasma of healthy participants with an intact colon approximately 6–12 h following the ingestion of supplements of chlorogenic acid, caffeic acid, or rutin [18,19,20]. Health-promoting effects have been related to the antioxidant capacity of plant foods, which has been partly attributed to (poly)phenolics [21]. However, the relationship of antioxidant activities of (poly)phenolics to their microbial by-products has not been well studied. Consequently, investigations regarding the identification and quantification of microbial colonic metabolites and their impact on antioxidant potential is highly relevant to the possible health benefits of (poly)phenolics.

An experimental approach to investigate the biotransformation of (poly)phenolics has involved simulated in vitro GI digestion models. Most GI model studies evaluating (poly)phenolic metabolism have focused on the enzymatic and chemical digestive conditions of the upper GI tract [22,23]. Alternatively, some studies have used a basic fermenter involving human or rat fecal incubations to assess microbial action on (poly)phenolics [24,25,26,27]. A more comprehensive approach that stimulates upper and lower GI digestion involves multistage in vitro models consisting of reactors pertaining to the stomach, SI, and the three colonic compartments of the ascending colon (AC), transverse colon (TC), and descending colon (DC) [28]. There have been limited studies of (poly)phenolic biotransformation involving a multistage GI stimulator. A recent study demonstrated that the feeding of pure reference (poly)phenolic compounds into a multireactor GI system led to microbial phenolic metabolite profiles and antioxidant activities that varied among the three colonic reactors [29]. Similarly, the multireactor-GI-stimulated digestion of red wine showed differing microbial phenolic metabolite profiles among the three colonic vessels [30].

In the present study, PRPE was subjected to digestion via a computer-controlled multistage GI model to examine phenolic catabolites in the colonic reactors using liquid chromatography-mass spectrometry (LC-MS). The antioxidant potential of the digests of PRPE was assessed at different stages of digestion using an antioxidant capacity assay.

## 2. Materials and Methods

### 2.1. Phenolic Extraction

PRPE was generated by POS Bio-Sciences (POS Bio-Sciences, Saskatoon, SK, Canada) as previously described [11]. In brief, 20 kg of “Onaway” potatoes were diced, freeze-dried, and extracted by agitation with 200 L of a 90% (*v*/*v*) aqueous ethanol solution for 1 h at room temperature. The ratio of powder to aqueous ethanol was 1:10 (*w*/*v*). After extraction, the extract was separated from the solids by centrifugation at 1076× *g* for 10 min. The extract was then concentrated under vacuum at 40–50 °C, until the volume was reduced to approximately 15 L and the ethanol percentage, measured using a hydrometer, was less than 10%. Water was added back during evaporation to attain a low ethanol content. Afterwards, the concentrate was freeze-dried to generate powdered PRPE with a phenolic content (mg/g) of chlorogenic acid (8.9), caffeic acid (0.6), ferulic acid (0.2), and rutin (1.2). PRPE was stored at −80 °C until use for the simulated GI model digestion studies. 

### 2.2. In Vitro Digestion of PRPE

In vitro digestion of PRPE in the computer-controlled gastrointestinal model followed a previously published method [28]. The GI model system involved five consecutive double-jacketed reactors, which represented the stomach, SI, AC, TC, and DC. The unit was fully computer-controlled using LabVIEW^®^ software (National Instruments, Austin, TX, USA), which controls the addition of food to the stomach, buffers for pH adjustment to the vessels, and pancreatic juice to the SI. The passage of food in the stomach was simulated by the addition of gastric solution (0.1 M HCl and pepsin; Sigma-Aldrich, Oakville, ON, Canada). The SI vessel was stimulated by addition of a pancreatic solution containing (12 g/L) NaHCO_3_ (S7277, Sigma-Aldrich, Oakville, ON, Canada), (6 g/L) Oxgall (DF0128-17-8, Fisher Scientific, Nepean, ON, Canada), and (0.9 g/L) pancreatin (P1750-100 g, Sigma-Aldrich). The flow of intestinal content between all reactors was automatically computer-controlled. The total transit time was 8 h, i.e., 2 h in the stomach and SI vessels, followed by 4 h in the colonic vessels. The total stabilization period was 2 weeks, whereby freshly collected fecal slurry samples were inoculated into the three colonic reactors. The fecal samples were obtained from five healthy volunteers having no history of GI disease or any use of antibiotics within the previous 6 months. During the stabilization period, the GI system was continuously fed three times per day with the GI nutrient solution. The GI nutrient solution was composed of (g/L): arabinogalactan (1), pectin (2), xylan (1), starch (3), glucose (0.4), yeast extract (3), peptone (1), mucin (4), and cysteine powder (0.5); as previously established to stabilize the microbial community in the colonic vessels [28]. Conditions in all the vessels of the simulated gut were kept strictly anaerobic by flushing nitrogen for 20 min into the air space daily and at the time of each manipulation. The nutritive media was stored at 4 °C until the time of injection. Temperature-controlled water flowed between the double-glass-jacketed reactors to keep the temperature at 37 °C. The pH in the vessels was continuously measured by pH probes and automatically adjusted using 0.2 M HCl (AC423795000, Fisher Scientific, Ottawa, ON, Canada) or 0.5 M NaOH solutions (415413, Sigma-Aldrich, Oakville, ON, Canada) to keep a pH of 2.0 in the stomach and 6.5 in the SI. The pH ranges in the reactors of the AC, TC, and DC were maintained at 5.60–6.40, 6.20–6.40, and 6.60–6.80, respectively.

After a 2-week stabilization period, 130 g of PRPE, containing 1.4 g of total (poly)phenolics, was administered into the gut model system. This amount is slightly above the average daily total (poly)phenolic intake of 1 g that is generally consumed daily through food or supplement intake [31]. The extract was dissolved using 25 mL of methanol and solubilized into the GI food as described in other studies involving the feeding of (poly)phenolic solutions to microflora [15,32]. The fluid in the vessels was continuously stirred using a magnetic stirrer and the vessels were wrapped with aluminum foil to prevent polyphenol photodecomposition. Digestion lasted 24 h and samples were taken from all the vessels before PRPE addition (*T* = 0 h) and every 8 h during the 24 h digestion. To ensure the stability of the polyphenols and inactivate digestive enzymes after the removal of digests, 5 mL of 0.5 M HCl solution was added to decrease the pH (pH < 2) of the withdrawn samples. The aliquots were centrifuged at 1000× *g* for 20 min and stored in 15 mL Falcon tubes at −80 °C for later analysis.

### 2.3. LC-MS Analysis of Phenolic Metabolites

Samples were thawed, vortexed, and filtered with 25 mm syringe filters (0.45 µm, MCE, sterile; Fisher Scientific Ottawa, ON, Canada) into 1 mL glass vials before analysis. Phenolic metabolites were separated and analysed using LC-MS analysis as previously described [29]. In brief, a Gemini-NX (5 µm, 100 mm × 4.6 mm) column (Phenomenex, Torrance, CA, USA) and a 4.6 mm × 2.0 mm guard column (Phenomenex, Torrance, CA, USA ) were used with two mobile phases: solvent A (10 mM formic acid, pH 3.5) and solvent B (5 mM ammonium formate solution in 100% methanol). The following gradient elution was used: 0 min 5% B, 2 min 5% B, 5 min 30% B, 7 min 70% B, 9 min 100% B, and 12 min 100% B, with a flow rate of 1.0 mL/min, and 20 µL of sample was injected. LC-MS analysis used a 6210 LC-MS Time of Flight system (Agilent Technologies, Santa Clara, CA, USA) in negative electrospray ionization mode with internal calibration using calibrant ions at *m/z* 119.0363 and 966.0007 at a scanning speed of 1 spectrum/s. Source conditions were as follows: capillary voltage of −4000 V, gas temperature of 350 °C, drying gas flow of 12 L/min, nebulizer gas at 50 psi, and fragmentor and skimmer voltages at 100 V and 60 V, respectively. The relative abundance of metabolized compounds was measured relative to the quantification of 3-hydroxyphenylacetic acid as the reference peak, as 3-hydroxyphenylacetic acid was noted to be present at the same concentration in all but the ascending colonic vessel at *T* = 0. 

### 2.4. Ferric Reducing Antioxidant Power (FRAP) Assay

The FRAP assay was used to determine the total antioxidant capacity of the sample through the reduction of the ferric tripyridyl triazine complex to a ferrous complex [33]. The FRAP reagents were prepared as previously described [34]. The reaction was carried out using a 96-well plate upon the addition of 30 μL H_2_O, 10 μL standards or samples, and 200 μL FRAP solution. Samples were left to react at room temperature for 20 min and absorbance was read at 593 nm in a microplate reader (Infinite PRO 200 series, Tecan Group, San Jose, CA, USA). Ferrous sulfate solution was used as an external standard with a calibration curve range of 0.1 to 10 mM. The results were expressed as ferrous sulfate equivalents. 

### 2.5. Statistical Analysis

Data were tested for normality and significant least square means (LSMeans) were compared using Tukey’s honest significant difference (HSD) posthoc test. Statistical significance was set at *p* < 0.05 and all statistical analyses were performed using SAS 9.2 (SAS Institute Inc., Cary, NC, USA). Data are expressed as means ± SEM.

## 3. Results and Discussion

### 3.1. Biotransformation of Polyphenols

Table 1 illustrates the profiles of the parent (poly)phenolics and their microbial metabolites in the three colonic vessels of the GI model at baseline (*T* = 0 h) and after 24 h digestion of PRPE. A total of 11 microbial metabolites were detected in the colonic vessels.

**Figure 1:**
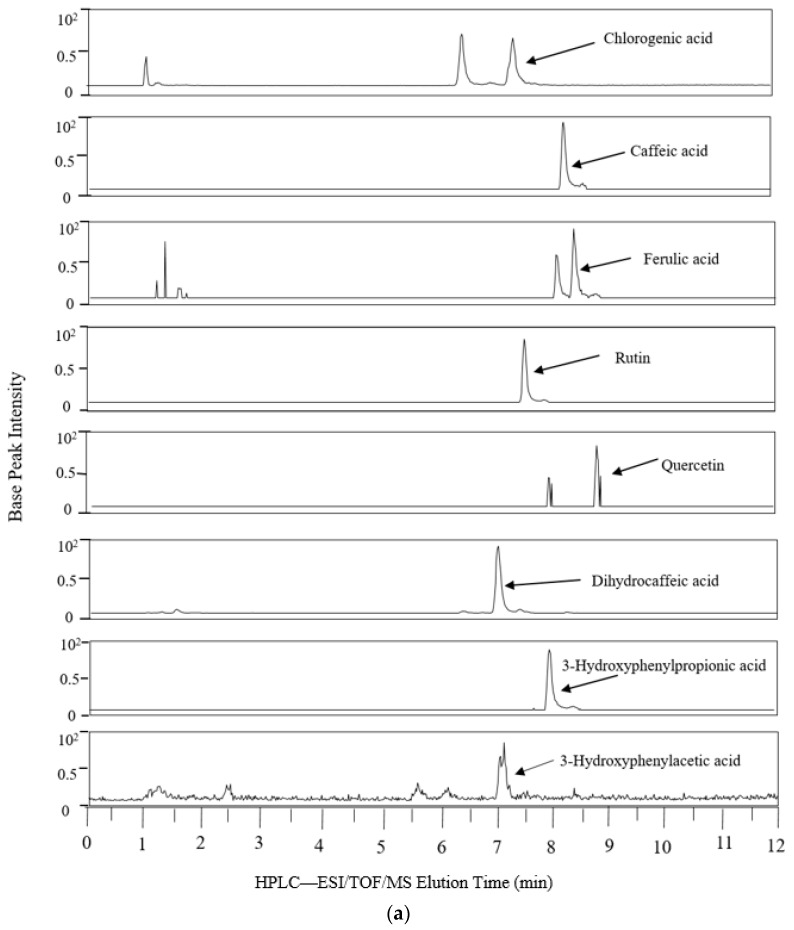

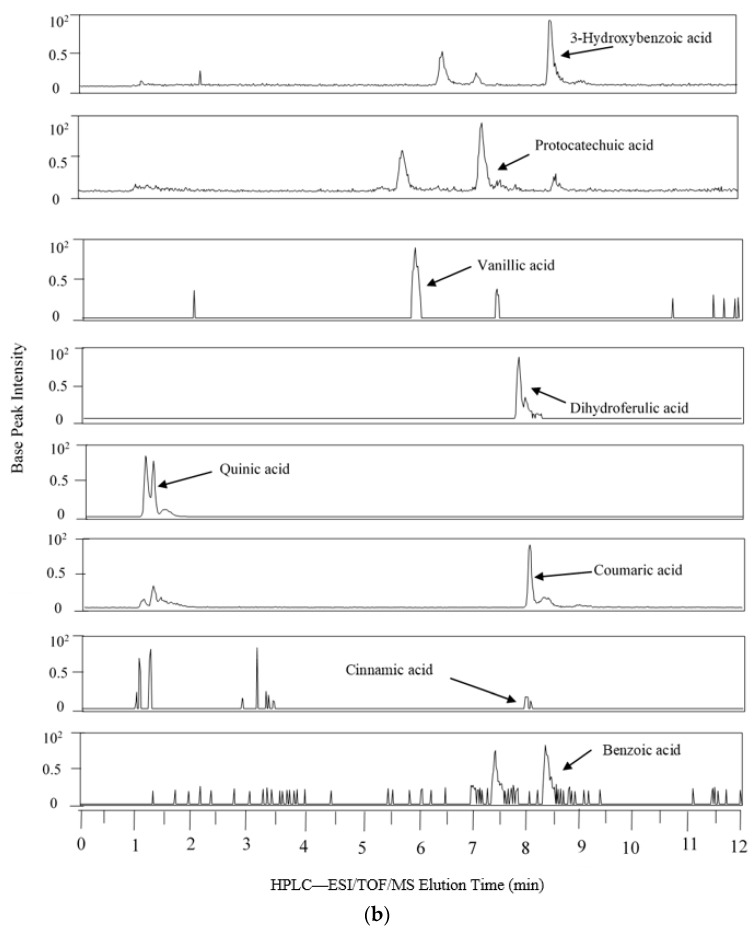
Representative extracted ion chromatograms from colonic vessels after 24 h of PRPE fermentation. HPLC-ESI/TOF/MS: High Performance Liquid Chromatography-Electrospray Ionization/Time of Flight/Mass Spectrometry. (**a**) AC; (**b**) AC continued.

Figure 1 shows the extracted ion chromatogram from the 24 h simulated digestion of PRPE in the AC compartment, which is a representative vessel that contained the native (poly)phenolics and their microbial metabolites. 

The phenolic profile differences among the colonic vessels could be attributed partly to different microbial communities, as the pattern of (poly)phenolic degradation has been previously noted to depend upon varying gut microbial profiles caused by the different pH range in each of the reactors [35]. Because the AC was the first reactor exposed to the parent (poly)phenolics, it contained relatively high levels of chlorogenic acid, caffeic acid, ferulic acid, and rutin in comparison with the other colonic vessels. Likewise, as parent (poly)phenolics begin to undergo microbial catabolism in the AC, this reactor accumulated the largest number of phenolic metabolites with greater relative abundance (except for 3-phenylpropionic acid) compared to the other colonic vessels. The major microbial catabolites produced from the fermentation of PRPE-derived (poly)phenolics included caffeic acid, dihydrocaffeic acid, phenylpropionic acid, 3-hydroxyphenylpropionic acid, 3-hydroxyphenylacetic acid, 3-hydroxybenzoic acid, and coumaric acid, which all have been detected in human fecal water and in fermentation studies using human fecal slurry [28,36]. The concurrent presence of native (poly)phenolics and their microbial metabolites in the AC following the 24 h digestion contrasts with batch fermentation studies with human fecal slurry showing the complete degradation of chlorogenic acid, caffeic acid, and rutin that can occur as early as 30 min to 5 h following incubation [24,25,37,38]. The high levels of quinic acid in the AC can be generated via bacterial cinnamoyl esterase-mediated cleavage of the ester bond between caffeic acid and quinic acid in chlorogenic acid [35,39,40]. The catabolism of chlorogenic acid would explain the high amounts of caffeic acid in the AC, in addition to the caffeic acid provided from PRPE itself. Caffeic acid undergoes microbial reduction of the double bond to generate dihydrocaffeic acid [32], which was also detectable at high levels in the AC. Dihydrocaffeic acid undergoes bacterial dehydroxylation to generate 3-hydroxylphenylpropionic acid, which was present at substantially lower levels in the AC; while 3-phenylpropionic acid, the microbial degradation product of 3-hydroxylphenylpropionic acid, was not detected. The above findings are in concert with Rechner et al. [32], who reported that larger doses of chlorogenic acid (500 to 1500 mg/L), similar to that provided in the present study, strongly shifted the composition of chlorogenic end-products to metabolites generated at the first catabolic steps. Therefore, high concentrations of caffeic and dihydrocaffeic acid generated from a large input of chlorogenic acid appear to inhibit their subsequent degradation to 3-hydroxylphenylpropionic acid and 3-phenylpropionic acid. Dihydrocaffeic acid can also be transformed via microbial α-oxidation or β-oxidation reactions to produce protocatechuic acid [32,38,41,42], detected in the AC. The PRPE supplement provided rutin, observed in the AC along with quercetin, a microbial degradation product of rutin [43], which was noted in low amounts. The presence of 3-hydroxylphenylpropionic acid and protocatechuic acid in the AC can be partly attributed to the bacterial degradation of quercetin [44]. The presence of ferulic acid in the AC can be obtained from the PRPE supplement as well as bacterial methylation of caffeic acid which can generate ferulic acid [45]. Ferulic acid can undergo further microbial metabolism to form vanillic acid [19], which was detected at low amounts in all the colonic reactors, indicating that this was a product of a relatively minor catabolic event. The parent (poly)phenolics provided by PRPE were absent in the TC and DC, apart from ferulic acid, which was seen at barely detectable levels (Table 1). Prior to PRPE addition, dihydrocaffeic acid, 3-hydroxyphenylacetic acid, 3-phenylpropionoic acid, cinnamic acid, and benzoic acid were measured in the colonic reactors. The presence of such catabolites has also been described in previous fecal fermentation studies prior to (poly)phenolic addition [29,30,35,38], and may be the result of protein and carbohydrate fermentation of the nutritive medium [30], as the two-week stabilization would likely have eliminated residual phenolics from the original fecal matter. The diminution of most of the microbial metabolites seen in the DC and TC was probably secondary to microbial β-oxidation reactions, which lead to the degradation of phenylacetic, phenylpropionic, and benzoic acids [46]. A major increase was seen in the levels of dihydrocaffeic acid in the DC compartment as compared to the relatively low levels in the TC, which suggests that further microbial phenolic biotransformation occurs in the DC. Overall, the detection of significant amounts of phenolic acids in the TC and DC coincides with previous multistage in vitro digestion models involving extracts of (poly)phenolic-rich red beverages such as wine, grape juice, and black tea [47,48]. 

### 3.2. Antioxidant Capacity

**Figure 2:**
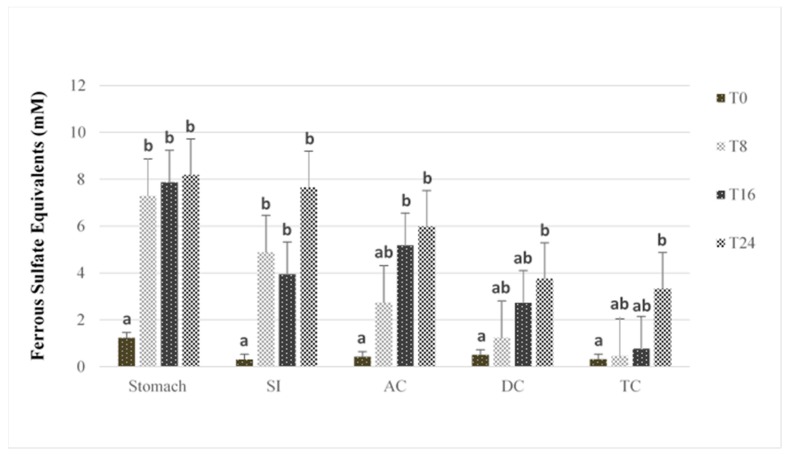
FRAP antioxidant capacity after human stimulated intestinal digestion at baseline (T0) and after 8 h (T8), 16 h (T16) and 24 h (T24) of digestion. SI = small intestine; AC = ascending colon; TC = transverse colon; DC = descending colon. Data are represented as means ± SE. Bars that do not share the same letter within the same vessel are significantly (*p* < 0.05) different from each other, based on Tukey’s honest significant difference (HSD) posthoc test.

Figure 2 shows the change in FRAP antioxidant capacity with time in the five compartments of the GI model after PRPE addition. 

In general, an increase in antioxidant activity was observed in all GI vessels upon the addition of PRPE, although the timing of the increase differed among the reactors. After 8 h of PRPE digestion, the stomach and SI reactors had significantly higher antioxidant capacity compared to the baseline (*p* < 0.05). In contrast, antioxidant capacity in the colonic vessels increased more slowly; this was greater after 16 h for the AC and 24 h for the DC and TC. As chlorogenic acid, caffeic acid, and rutin have antioxidant properties [43,49], the more rapid increase in antioxidant capacity in the stomach and SI reactors can be attributed to the parent (poly)phenolics, which showed no apparent degradation in those vessels (see Supplementary Material). Similarly, the relatively shorter time for increased antioxidant capacity following PRPE addition in the AC compartment versus the DC and TC can be attributed to the concurrent presence of both the parent (poly)phenolics and their microbial metabolites in the AC. Hence, microbial catabolism of the parent (poly)phenolics appeared to initially reduce antioxidant activity, which agrees with previous findings involving the fecal microbial catabolism of (poly)phenolic compounds [13,29]. On the other hand, the increase in antioxidant capacity observed at 24 h in the DC and TC can be linked to the accumulation of microbial smaller-molecular-weight phenolic catabolites, as the parent (poly)phenolics had completely degraded in those vessels. This finding coincides with that of an earlier study showing increased antioxidant capacity with the generation of microbial phenolic metabolites after the degradation of pure reference (poly)phenolics using an identical in vitro digestion model [29]. Several studies have reported effective antioxidant properties in relation to the microbial phenolic metabolites detected in the present study, including coumaric acid, 3-hydroxyphenylpropionic acid, vanillic acid, dihydrocaffeic acid, and protocatechuic acid [29,50]. 

## 4. Conclusions

In conclusion, the association of antioxidant activities with the simpler microbial phenolic acid metabolites generated from PRPE digestion highlights the utility of simulated digestion and fermentation for the assessment of bioactive properties of polyphenol-rich plant food extracts. Further in vivo investigations and mechanistic studies are needed to determine the physiological relevance of the free-radical-scavenging activities associated with the microbial metabolites of potato-derived polyphenols.

## Figures and Tables

**Table 1:** Polyphenols and their metabolites after human-stimulated intestinal digestion at baseline (T0) and after 24 h (T24) postdigestion ^1^.

Theoretical Mass (*m*/*z*) ^2^	Measured Mass	Mass Accuracy (ppm)	Retention Time (min)	Common Name	Systematic Name	AC		TC		DC	
						T0	T24	T0	T24	T0	T24
609.1461	609.1422	6.4	8.7	Rutin	Quercetin-3-*O*-rutinoside	-	9.62	-	-	-	-
353.0878	353.0863	4.3	7.5	Chlorogenic acid	(1*S*,3*R*,4*R*,5*R*)-3-{[(2*E*)-3(3,4-Dihydroxyphenyl)prop-2enoyl]oxy}1,4,5trihydroxycyclohexanecarboxylic acid	-	37.96	-	-	-	-
301.0354	301.0395	13.7	8	Quercetin	2-(3,4-Dihydroxyphenyl)-3,5,7-trihydroxy-4H-chromen-4-one	-	1.05	-	-	-	0.15
193.0506	193.0499	3.8	8.5	Ferulic acid	3-(4-Hydroxy-3-methoxy-phenyl)prop-2-enoic acid	-	2.03	-	0.16	-	-
191.0561	191.0544	2.3	1.7	Quinic acid	(1S,3R,4S,5R)-1,3,4,5-Tetrahydroxycyclohexanecarboxylic acid	-	642.68	-	28.74	-	1.39
181.0506	181.0505	0.7	7.7	Dihydrocaffeic acid	3- (3′,4′-Dihydroxyphenyl) propionic acid	-	136.83	36.16	2.92	13.70	37.05
179.0325	179.339	6.1	8	Caffeic acid	3,4-Dihydroxycinnamic acid	-	327.36	-	-	-	0.50
167.035	167.0346	2.2	6.6	Vanillic acid	4-Hydroxy-3-methoxybenzoic acid	-	0.10	-	0.03	-	0.02
165.0557	165.0557	0.1	8.4	3-Hydroxylphenyl propionic acid	3-(3′-Hydroxyphenyl)propionic acid	-	36.69	-	28.59	-	7.56
163.0401	163.0401	0.2	8.4	Coumaric acid	The isomer is not specified from our data	-	12.34	-	1.05	-	-
153.0193	153.0204	6.9	7.2	Protocatechuic acid	3,4-Dihydroxybenzoic acid	-	3.65	-	3.26	-	2.64
151.0401	151.0409	5.5	7.7	3-Hydroxyphenyl acetic acid	3-Hydroxyphenylacetic acid ^3^	-	1.00	1.00	1.00	1.00	1.00
149.0608	149.0604	2.7	9.4	3-Phenylpropionic acid	3-Phenylpropionoic acid	-	0.00	5.01	1.55	1.53	1.04
147.0452	147.0453	1	8.5	Cinnamic acid	3-Phenylprop-2-enoic acid	-	0.74	0.46	-	-	-
137.0244	137.0245	0.6	7.2	3-Hydroxybenzoic acid	3-Hydroxybenzoic acid	-	9.02	-	8.88	-	3.03

AC = ascending colon; TC = transverse colon; DC = descending colon; (−) absent; masses are shown as [M − H]. ^1^ Determined by liquid chromatography-mass spectrometry (LC-MS) analysis. ^2^ Identification based on previous literature [19,25,32]. ^3^ The quantities of the polyphenols and their metabolites are calculated relative to the concentration of 3-hydroxyphenyl acetic acid.

## Supplementary Materials

The following are available online at http://www.mdpi.com/2076-3921/7/3/43/s1. The supplementary material file includes data acquired via HPLC depicting the catabolism of parent (poly)phenolics and appearance of metabolites throughout the five vessels of the GI model after 24 h in vitro digestion, in two figures (Figures S1 and S2). The methodology for acquisition of HPLC data is also described. Figure S1: HPLC Chromatograms of (poly)phenolics in colonic vessels at baseline and after 24 h of fermentation. Figure S2: HPLC Chromatograms of polyphenols in (A) digested potato extract, (B) stomach vessel (V1), and (C) small intestine vessel (V2) after 24 h of digestion.
