# Supplementary material for: Microbial Biotransformation of a Polyphenol-Rich Potato Extract Affects Antioxidant Capacity in a Simulated Gastrointestinal Model
Source: Antioxidants (Basel). 2018 Mar 20;7(3):43. doi: 10.3390/antiox7030043 (PMC5874529; doi:10.3390/antiox7030043)
Supplement: Supplementary file 1 [file antioxidants-07-00043-s001.zip › Antioxidants - Kubow - Supplementary Material.docx]

**Microbial Biotransformation of a Polyphenol-Rich Potato Extract Affects Antioxidant Capacity in a Simulated Gastrointestinal Model**

**Supplementary material**

**Materials and Methods: High Performance Liquid Chromatography (HPLC) Identification of (poly)phenolic compounds**

Samples were thawed with UV filtered light systems, vortexed, and filtered using 25 mm Syringe Filters (0.45 µm, MCE, sterile) (Fisher Scientific Ottawa, ON) into 1 mL glass vials before HPLC injection. Samples were kept chilled at all times and shielded from bright light. The HPLC identification of a compound was based on the retention time as compared to that of purchased pure standards (CGA C3878, CA C0625, FA 12,870 and RU R5143; Sigma-Aldrich, St. Louis, MO) and verified by LC-MS. A Varian HPLC system equipped with a Varian 9012 tertiary pump module, refrigerated auto-sampler model 410 and single variable wavelength detector module 9050 operated by a Varian Star 5.3 software was used for sample analysis. Phenolic compounds were separated based on a modified method^1^, using a reverse phase HPLC Gemini-NX (5 μm, 100 mm × 4.6 mm) column (Phenomenex, Torrance, CA, USA) and a 4.6 mm × 2.0 mm guard column. Two buffers were used as mobile phases. Buffer A was a 10 mM formic acid solution prepared by dissolving 0.4603 g of formic acid in 1 L distilled water. The pH was adjusted at 3.5 using 1 M NH_4_OH solution. Buffer B was a 5 mM ammonium formate solution prepared by dissolving 0.3153 g of ammonium formate in 1 L 100 % methanol and stirred on a magnetic stirrer. The solvent gradient was as follows: 0-1 min 100 % buffer A, 1-5 min 0-30 % buffer B, 5-8.5 min 30-70 % buffer B, 8.5-12 min 70-100 % buffer B. UV detection was conducted at 320 nm. A flow rate of 1.0 mL/min was used and 20 μL of sample were injected. Samples were analyzed in duplicate.

**Figure Captions**

**Figure S1.** HPLC Chromatograms of (poly)phenolics in colonic vessels at baseline and after 24 h of fermentation. Polyphenolic profile of (A) vessels at baseline, (B) PRPE after digestion in V3 (ascending colon), (C) V4 (transverse colon), and (D) V5 (descending colon): (1) neocholorogenic acid, (2) chlorogenic acid, (3) caffeic acid, (4) ferulic acid, (5) rutin, (x) tryptophan, (ᵿ) quinic acid, (γ) cryptoCGA (ẅ) dihydrocaffeic acid, (ℓ) 3-Hydroxyphenylpropionic acid, (*) new peak formed upon microbial biotransformation of polyphenols.

**Figure S2.** HPLC Chromatograms of polyphenols in (A) digested potato extract, (B) stomach vessel (V1), and (C) small intestine vessel (V2) after 24 h of digestion: (1) neocholorogenic, acid (2) cholorogenic acid, (3) caffeic acid, (4) ferulic acid, (5) rutin, (x) tryptophan, (γ) cryptochlorogenic acid.

**References**

1. Shakya, R. and Navarre, D. Rapid screening of ascorbic acid, glycoalkaloids, and phenolics in potato using high-performance liquid chromatography*.* *J Agr Food Chem*. **2006**, 54, 5253-5260.

**Figure** **S1**

0

100

50

150

200

10.0

2.5

5.0

7.5

12.5

Absorbance at 280 nm

A

B

Absorbance at 280 nm

3

5.0

2.5

0

7.5

12.5

10.0

*

ℓ

50

100

125

75

150

x

ᵿ

*

*

1

*

*

*

4

5

γ

*

ẅ

*

*

2

25

*

Retention Time (minutes)

12.5

7.5

ℓ

ẅ

*

*1

*

*

*

**

*

*

5.0

2.5

x

25

0

100

50

75

*

ᵿ2

*

10.0

Retention Time (minutes)

C

D

Absorbance at 280 nm

7.5

*

*

*

5.0

10.0

12.5

ẅ

*

*

*

*

*

x

ℓ

2.5

75

0

25

50

100

ᵿ

*

*

Absorbance at 280 nm

Retention Time (minutes)

Retention Time (minutes)

**Figure S2**

A C

3

2

1

12.5

10.0

2.5

5.0

7.5

200

300

4

γ

400

0

100

x

5

Absorbance at 280 nm

2

1

3

4

7.5

5.0

2.5

300

0

400

200

100

10.0

12.5

x

5

Absorbance at 280 nm

Retention Time (minutes)

Retention Time (minutes)

B

10.0

7.5

2.5

5.0

12.5

500

100

800

600

400

300

700

200

0

1

γ

3

4

5

2

x

Absorbance at 280 nm

Retention Time (minutes)
